# Assessment of the difficulties in laparoscopic cholecystectomy among patients at Baghdad province^☆^

**DOI:** 10.1016/j.amsu.2019.03.008

**Published:** 2019-03-27

**Authors:** Basim Rassam Ghadhban

**Affiliations:** Department of Surgery, University of Baghdad/College of Medicine, Baghdad, Iraq

**Keywords:** Laparoscopic cholecystectomy, Difficulty score.BMI, Admission

## Abstract

**Background:**

Laparoscopic surgery for gallbladder cholecystectomy has become the typical procedure for symptomatic gallbladder stone diseases treatment as a type of minimal invasiveness surgery associated with less pain and early recovery as there is minimal trauma of access without shrinking the exposure of operative field. The current study aimed to assess factors associated with difficult LC.

**Methodology:**

A cross sectional study was conducted at medical city complex (Baghdad Teaching Hospital) from October 2015 to October 2016, in which all patients that were admitted for LC were examined preoperatively, underwent LC and followed postoperatively to study factors associated with difficult LC. All LC were operated by qualified senior surgeons and supervised by well-trained resident doctors. A pre-operative score system parameters were obtained from history, clinical examination and investigation findings.

**Results:**

Laparoscopic cholecystectomy was performed for 100 patients, 78% were females and 49% were among 40–59 years of age. The preoperative scoring revealed that in 58% of the patients the score indicate difficulty, and very difficult in only 7% of the patients. Difficult and very difficult operations were significantly associated with high difficult scoring preoperatively, gender, BMI and cause of admission.

**Conclusion:**

High difficult scoring preoperatively, gender, BMI and cause of admission can be used as predictors for difficult LC.

## Highlights

• The current study aimed to assess factors associated with difficult LC.
• Very difficult operations were associated with high difficult scoring preoperatively, gender, BMI and cause of admission.
• Laparoscopic surgery for gallbladder cholecystectomy has become the typical procedure for symptomatic cholelithiasis treatment.

## 1 Introduction

Cholecystectomy by Laparoscopic surgery affords an effective and safe model treatment for many patients with symptomatic gallbladder stone diseases [1] and is the golden treatment of choice for cholelithiasis. It has now become the most common operation performed in United States has increased from 5 to 7 lakhs/year [2]. The rewards of laparoscopic cholecystectomy are earlier reappearance to bowel function, less postoperative pain, cosmetics, shorter length of hospital stay, earlier yield to full activity, and decreased overall cost [3,4]. The purpose of this study is to determine the predictive factors for difficult laparoscopic cholecystectomy.

Laparoscopic cholecystectomy has become the typical surgery for treatment of symptomatic cholelithiasis as a type of minimal invasiveness surgery associated with less pain and early recovery as there is minimal trauma of access without shrinking the exposure of operative field, in fact it has the advantage of better exposure and a better view [5]. Since the starter in 1989, cholecystectomy by laparoscopic surgery had been the standard surgery in the treatment of symptomatic gall stone but some of the scheduled LC needs conversion due to numerous factors [6].

Sometimes LC becomes difficult and takes more than expected to complete the surgery even when there are bile or spillage of stone and sometime it requires conversion to open cholecystectomy in order to safely finish the operation [7].

The grade of difficulties is difficult to expect but it is important to be estimated for both the surgeon to be ready to deal with a difficult case and the patient to be informed about the possibility of conversion and get adequate explanation [8].

It's be worthy to have dependable prognostic factors for alteration or obstacles in Lap cholecystectomy. Patients can then be designated for ambulatory surgery or admission, and cases with high risk can be advised of high chances of conversion and potential complications. The preparation of operating team can be achieved to perform operative cholangiograms or to change to OC if the need arises [9] and the cost of the operation can be estimated more clearly [10].

### 1.1 The aim

The aim of this study was to define preoperative indicators that can be used for expecting conversion to OC.

## 2 Methods

A cross sectional study was achieved at Medical city(Baghdad Teaching Hospital) from October 2015 to October 2016, in which all patients that were admitted for LC. Proper history was taken from all patients with thorough physical examination. Abdominal ultrasonography (USG), Complete Blood Count (CBC), Renal and liver function tests were performed for all the patients, ERCP and MRCP were done only in selective cases.

Patients with Chronic liver disease, CBD stone and features of obstructive jaundice were excluded from the study.

On day of admission before operation, each patient was scored according to the eleven parameters which were by history, Age, Sex, previous admission to hospital for acute cholecystitis or biliary colic and by clinical examination including Body mass index (BMI), Abdominal scar, Palpable gallbladder and radiological findings “Wall thickness, Pericholecystic collection, Impacted stone”. Accordingly patients were put in two groups, easy with score ≤5 and difficult with score >5.

All LC were operated by qualified senior surgeons and supervised by well-trained resident doctors.

Surgery were done by using CO2 pneumoperitoneum with 10–12 mm Hg pressure and using standard two 10 mm and two 5 mm ports.

The timing was started from the first port site incision till the last port closure. All the intraoperative events were documented. Operations were done under general anesthesia.

Postoperatively all cases were again assorted into easy and difficult groups, easy, if duration of surgery was ≤60 min, no injury to common bile duct, and no conversion to open, and difficult, if duration >60 min, conversion to OC and/or injury to common bile duct.

All cases received standard postoperative care and follow up. Drain was removed in either the first or the second postoperative day according to the amount of drainage. Suture removal was done on the 7th and 10th postoperative day.

### 2.1 Ethical Consideration

Formal consent was gotten from the ethical committee of the Department of surgery, We explained The aim and details of surgery to the patients and a verbal consent was obtain before including the patient.

### 2.2 Statistical analysis

Data was coded and analyzed using Microsoft Excel 2010. Continuous variables were presented as mean ± Standard Deviation (SD) and categorical variables were presented as frequency and relative frequency. Student T test was used to test the significant differences between means and Chi squared test was used to test the significant association between categorical variables. P value of <0.05 was considered statistically significant.

## 3 Results

One hundred patients of laparoscopic cholecystectomy patients were enrolled in the current revision, 78 females and 22 males (with a female to male ratio of 3.5:1)Their age ranged from 17 to 70 yrs with a mean of 43.5 ± 13.2 yrs Standards Deviation (SD) and a median of 43. Although the males were older (mean age 46.3 ± 14.8 SD) than the females (42.8 ± 12.7 SD) yet the difference in mean age was statistically not significant (Student's t-test, df = 98(t_(0.975)_), P = 0.27).

Classifying the patients into age groups showed that 49% of them were among 40–59 years of age, 38% were below 40 and only 13% were aged 60 and above (Table 1).

**Table 1:** Distribution of patients by age groups (in years), Body Mass Index (BMI), Difficulty Scores, Operation and cause of admission.

Characteristics	Patients (100)	
	No.	%
Age groups (in years)		
<40	38	38.0
40–49	30	30.0
50–59	19	19.0
≥60	13	13.0
BMI		
<25	18	18.0
25–29.9	69	69.0
≥30	13	13.0
Difficulty score		
Easy (≤5)	55	55.0
Difficult (>5)	45	45.0
Operation		
Easy	48	48.0
Difficult	43	43.0
Very difficult	9	9.0
Cause of admission		
Cholecystitis	63	63.0
Biliary colic	27	27.0
G. S. Pancreatitis	7	7.0
Polyp	3	3.0

Regarding BMI; 69% were overweight and 13% were obese (Table 1), the majority of both were females with a statistically significant association (χ^2^ = 10.03, df = 2, P = 0.006) (Table 2) (see Table 3, Table 4).

**Table 2:** Cross tabulation of age groups (in years), Body Mass Index (BMI), Difficulty score and cause of admission by gender.

Characteristics	Males (22)		Females (78)		Total (100)		P value
	No.	%	No.	%	No.	%	
Age groups (in years)							
<40	7	18.4	31	81.6	38	100.0	0.49
40–49	6	20.0	24	80.0	30	100.0	
50–59	4	21.1	15	78.9	19	100.0	
≥60	5	38.5	8	61.5	13	100.0	
BMI							
<25	9	50.0	9	50.0	18	100.0	0.006*
25–29.9	11	15.9	58	84.1	69	100.0	
≥30	2	15.4	11	84.6	13	100.0	
Difficulty score							
Easy (≤5)	9	16.4	46	83.6	55	100.0	0.1
Difficult (>5)	13	28.9	32	71.1	45	100.0	
Operation							
Easy	5	10.4	43	89.6	48	100.0	0.01*
Difficult & Very difficult	17	32.7	35	67.3	52	100.0	
Cause of admission							
Cholecystitis	13	20.6	50	79.4	63	100.0	0.8
Biliary colic	6	22.2	21	77.8	27	100.0	
Others(gall blader thickness)	3	30.0	7	70.1	10	100.0	

Taking the difficulty score in consideration revealed that high scores were significantly associated with difficult and very difficult operations (χ2 = 25.7, df = 2, P < 0.001), and with cause of admission (χ2 = 14.5, df = 2, P = 0.001) and that age, gender and BMI failed to reach statistically significant association (Table 2).

**Table 3:** Cross tabulation of difficulty score, gender, age groups (in years), Body Mass Index (BMI), and cause of admission by Type of operation (Easy vs. Difficult and very difficult).

Variables	Easy operation		Difficult & Very Difficult Operation		Total		P value
	No.	%	No.	%	No.	%	
Difficulty Score							
Easy	39		16		55		<0.001*
Difficult & Very difficult	9		36		45		
Gender							
Males	5		17		22		0.007*
Females	43		35		78		
Age groups (in years)							
<40	22		16		38		0.26
40–49	12		18		30		
50–59	10		9		19		
≥60	4		9		13		
BMI							
<25	8		10		18		0.03*
25–29.9	38		31		69		
≥30	2		11		13		
Cause of admission							
Cholecystitis	26		37		63		0.01*
Biliary colic	18		9		27		
G.S.Pancreatitis	1		6		7		
Polyp	3		0		3

**Table 4:** Cross tabulation of Laboratory Investigations and Sonography results.

Laboratory Investigations		score	max. score	patients score
S. Alkaline Phosphatase	>144 IU/L	1	1	28
	<144 IU/L	0		72
WBC Count	>11,000	1	1	33
	<11,000	0		67
Sonography results				
Gall bladder wall thickness	Thin <4 mm	0	2	72
	Thick ≥4 mm	2		18
Pericholecystic collection	NO	0	1	86
	YES	1		14
Impacted stone	NO	0	1	91
	YES	1		9
			Total score: 17	

According to the difficulty score; 55% of the patients were with easy score and 45% were with difficult score (Table 1), mostly were females, yet the association between gender and difficulty score was statistically not significant (χ^2^ = 2.2, df = 1, P = 0.1) (Table 2).

Operation was easy going in 48% of patients, difficult in 43%, and very difficult in 9% of patients (Table 1) with female predominate reaching a statistically significant association (χ^2^ = 7.2, df = 1, P = 0.01) (Table 2).

Nearly two thirds of the patients (63%) were admitted as a case of cholecystitis, 27% as Biliary Colic, 7% as G. S. Pancreatitis and only 3% as polyp (Table 1), still the association with gender was statistically not significant (Table 2).

## 4 Discussion

Hundred laparoscopic cholecystectomy patients which included in the present study revealed that females were most frequently than males the results was in agree with.

Torres [11] reported that Female gender is a risk factor than male other study by.

George Bazoua [12] who documented gender affects the duration of surgery because more time is required to complete LC in men than in women.

We also reported that age 40 and less were most underwent laparoscopic cholecystectomy, This finding was not agree with [13] who decided underwent laparoscopic cholecystectomy, that for age of patients more than 40 and age of less than 30.

Operating surgeons had found were anticipated was easy to perform operations in cases, that was proposed a difficult surgery this was agree with [14]who found in their study the mainstream of the incidents and complications which may occur postoperative were related to(the occurrence of an acute cholecystitis) and were in part due to (some technical limits of the laparoscopic technique of the gallbladder bed peritonisation). The minimally invasive treatment of postoperative complications, was very effective and obtainable optimum healing conditions.

In our study, it was noticed that gall bladder wall thickness is an important factor in predicting difficulty as well as stone impaction in the neck of the gallbladder, which is gallbladder wall thickness by ultrasonography, ALP elevation, and increase WBC count determine conversion Gall bladder wall thickness on preoperative USG represents the presence of inflammation or fibrosis due to previous assaults of chlecystitis Previous admission for gall bladder disease was also significant predictor factors of difficulty In the other study, found attacks of cholecystitis, ultra-sonographic finding including thickness of GB, size and number of stones in GB encountered during surgery. In the current study, the age was noticed to be more higher in patients with difficult laparoscopic cholecystectomy in comparison to other group of non-difficult laparoscopy [15].

BMI was important factor in our study. In other study by Lee et al. [16] a negative relationship between BMI and the inflammation degree of cholecystitis in males, which resulted in a higher incidence of severe cholecystitis in the no obese male patients who not agreement with this study.

Pericholecystic collection was not significant although it is a marker for acute state the result was agree with [17]. Other studies revealed that preoperative US can help expect the operative difficulty of LC.

According to US findings, surgeon scan choose the cases appropriate for their skills, targeting to reducing operative complications and decreasing the waste of time.However, this preoperative US evaluation was not completely reliable in all patients, as already pointed out by other authors [18,19].

Bethany [20] suggested other clinical processes including medical history (abdominal scarring and previous abdominal surgery) and physical examination (palpable gallbladder and abdominal tenderness) were important patient factor predictors of a difficult procedure of cholecystectomy [21].

Ultrasonographic finding (thickness of GB, size and number of stones in GB encountered during surgery). In current study, age was noticed to be higher in patients with difficult laparoscopic cholecystectomy in comparism to group with non-difficult laparoscopy.

In few studies, patients with (previous abdominal surgery) is the factor that estimate difficult laparoscopic cholecystectomy and change to open cholecystectomy. In always of the studies(gall bladder wall thickness) has been considered as a significant factor for conversion. In present study, gall bladder wall thickness of 3 mm was found to be critical.Hutchinson et a l [22], Kama et al. [23] and Nachani [24].

To the best of our familiarity, this study is the first report assessing predictive factors for prediction of difficult laparoscopic cholecystectomy in Iraq.

## 5 Conclusion

High difficult scoring preoperatively, gender, BMI and cause of admission can be used as predictors for difficult LC, gall bladder wall thickness has been identified as a risk factor of LC in addition to Pericholecystic collection and Impacted stone.

## Ethical approval

**Ethical Consideration.** Official approval was gotten from the ethical committee of the hospital.

The aim of the study and the operation in details was explained to the patients and a verbal consent was obtain before including the patient.

## Author contribution

By:Ass. Prof. Dr. Basim Rassam Ghadhban With help from University of Baghdad/Baghdad College Of Medicine/Department of Surgery.

## Conflicts of interest

There is no conflicts of interest.

## Trial registry number

https://www.researchregistry.com/

researchregistry4375.

## Guarantor

Ass. Prof. Dr. Basim Rassam Ghadhban.

## Provenance and peer review

Not commissioned, externally peer reviewed.

## Sources of funding

There is no sources of funding for your research.
